# Taekwondo Fighting in Training Does Not Simulate the Affective and Cognitive Demands of Competition: Implications for Behavior and Transfer

**DOI:** 10.3389/fpsyg.2018.00025

**Published:** 2018-01-31

**Authors:** Michael A. Maloney, Ian Renshaw, Jonathon Headrick, David T. Martin, Damian Farrow

**Affiliations:** ^1^Institute of Sport, Exercise and Active Living, Victoria University, Melbourne, VIC, Australia; ^2^Movement Science Department, Australian Institute of Sport, Canberra, ACT, Australia; ^3^School of Exercise and Nutrition Sciences, Queensland University of Technology, Brisbane, QLD, Australia; ^4^School of Allied Health Sciences, Griffith University, Gold Coast, QLD, Australia; ^5^Philadelphia 76ers, Philadelphia, PA, United States

**Keywords:** affective learning design, ecological dynamics, representative learning design, representative design, transfer, Taekwondo

## Abstract

Enhancing practice design is critical to facilitate transfer of learning. Considerable research has focused on the role of perceptual information in practice simulation, yet has neglected how affect and cognition are shaped by practice environments and whether this influences the fidelity of behavior (Headrick et al., 2015). This study filled this gap by examining the fidelity of individual (cognition, affect, and actions) and interpersonal behavior of 10 highly skilled Australian Taekwondo athletes fighting in training compared to competition. Interpersonal behavior was assessed by tracking location coordinates to analyze distance-time coordination tendencies of the fighter–fighter system. Individual actions were assessed through notational analysis and approximate entropy calculations of coordinate data to quantify the (un)predictability of movement displacement. Affect and cognition were assessed with mixed-methods that included perceptual scales measuring anxiety, arousal, and mental effort, and post-fight video-facilitated confrontational interviews to explore how affect and cognitions might differ. Quantitative differences were assessed with mixed models and dependent *t*-tests. Results reveal that individual and interpersonal behavior differed between training and competition. In training, individuals attacked less (*d* = 0.81, *p* < 0.05), initiated attacks from further away (*d* = -0.20, *p* < 0.05) and displayed more predictable movement trajectories (*d* = 0.84, *p* < 0.05). In training, fighters had lower anxiety (*d* = -1.26, *p* < 0.05), arousal (*d* = -1.07, *p* < 0.05), and mental effort (*d* = -0.77, *p* < 0.05). These results were accompanied by changes in interpersonal behavior, with larger interpersonal distances generated by the fighter–fighter system in training (*d* = 0.80, *p* < 0.05). Qualitative data revealed the emergence of cognitions and affect specific to the training environment, such as reductions in pressure, arousal, and mental challenge. Findings highlight the specificity of performer–environment interactions. Fighting in training affords reduced affective and cognitive demands and a decrease in action fidelity compared to competition. In addition to sampling information, representative practice needs to consider modeling the cognitions and affect of competition to enhance transfer.

## Introduction

A key issue for practitioners working in competitive sport is enhancing the design of practice to facilitate the transfer of skills from training to competition. One way to enhance practice is through simulating key aspects of competition through the design of representative learning tasks (Araujo et al., 2007; Pinder et al., 2011b; Barris et al., 2014). However, recent theorizing has highlighted that designing adequate simulations of competitive performance environments in practice is not simple and requires consideration of factors other than information and action (Oudejans and Pijpers, 2010; Headrick et al., 2015). For example, in competition performers must adapt to unique constraints such as consequences, prizes, referees, crowds, and unfamiliar opponents, given the complexity of performance environments and an acknowledgment of the impact that affect^1^ and cognitions may have on perceptions and actions in high stakes competition, representative practice tasks need to also model the cognitive, affective, and behavioral demands of competition (Pijpers et al., 2006; Headrick et al., 2015). However, currently there is little understanding of the extent to which typical training environments adequately simulate the affective and/or cognitive demands of competition and whether this impacts on the fidelity of training behavior and subsequent transfer. Therefore, the paper aims to explore this issue in a combat sport setting and assess whether Taekwondo fighting in training adequately simulates the affective and cognitive demands of competition, and subsequently, whether the affective-cognitive demands observed in training impact on the representativeness of individual and interpersonal behavior relative to competition. A growing body of work has explored how improving training task design can potentially enhance the learning and transfer of skills to competition environments (Araujo and Davids, 2015). One way to describe the usefulness of different training tasks in sport is through the lens of ecological dynamics (Davids and Araújo, 2010). Ecological dynamics integrates concepts from dynamical systems theory and ecological psychology to understand how athletes coordinate their actions with the surrounding environment (Brunswik, 1956; Gibson, 1979; Kelso, 1995). An underpinning principle of this approach is the need for learners to form functional relationships with their environment (Fajen and Warren, 2003; Fajen et al., 2009).

In sport specific environments, social and physical information supports athletic behavior and provide opportunities for action (Fajen et al., 2009; Rietveld and Kiverstein, 2014). As behavior is regulated prospectively by a continuous process of perceiving and moving, invitations for action emerge in the form of affordances as an athlete moves around the environment picking up information (Gibson, 1979). For instance, in the combat sports, picking up certain postural or kinematic information from an opponent might invite an opportunity to attack. As an athlete learns, they attune to environmental features and the different actions they afford (Gibson, 1979; Bruineberg and Rietveld, 2014). Attunement ‘educates’ the attention of performers toward the most useful information, improving their ‘fit’ within the environment (Michaels and Jacobs, 2007; Bruineberg and Rietveld, 2014). The implications for training design in sport are that the coupling between the performer and environment present in competition needs to be preserved so that athlete learnings can transfer between environments.

These implications were captured by marrying concepts from Gibson’s ecological psychology and Brunswik’s representative design to develop a framework to guide the design of practice environments in sport (Brunswik, 1956; Gibson, 1979; Pinder et al., 2011b). Representative learning design emphasizes the need for the practice task constraints to represent the task constraints of the competition task (Pinder et al., 2011a). Therefore, any practice needs to satisfy this principle if transfer from practice to competition is to be optimized. A way to evaluate the potential for practice to transfer is through the specificity of relationship between performer and environment. The specific nature of this relationship – our actions are tightly coupled to specific information – provides a principled approach for scientists to evaluate the representativeness of different training tasks through comparing the fidelity of action responses (Stoffregen et al., 2003; Pinder et al., 2011b; Davids et al., 2012; Araujo and Davids, 2015).

Action fidelity refers to the correlation between a performance in a reference situation (real world environment) and a performance in a simulated situation (e.g., training) (Stoffregen et al., 2003; Stoffregen, 2007; Pinder et al., 2011b). The concept of fidelity specifically deals with transfer and is assessed in terms of task performance. Fidelity is achieved when behavior in a simulated (e.g., training) task represents the behavior observed in the performance task (Stoffregen, 2007). The fidelity of athlete behavior in learning tasks is known to be impacted when practitioners omit key ecological constraints to create *non-representative* practice conditions (Shim et al., 2005; Pinder et al., 2009; Dicks et al., 2010; Barris et al., 2013; Greenwood et al., 2016). For example, when cricket batters practiced with a ball projection machine as opposed to a human bowler it resulted in re-organized low fidelity action responses (Pinder et al., 2011a). In contrast, fidelity is maintained when practitioners sample key informational constraints from performance environments to design *representative* practice tasks (Dicks et al., 2010; Pinder et al., 2011a; Barris et al., 2013; Greenwood et al., 2016). Designing representative practice tasks that maintain fidelity will theoretically have positive implications for transfer (Brunswik, 1956; Araujo et al., 2007; Pinder et al., 2011b; Araujo and Davids, 2015). However, much of this research has focused on the utility of different external information sources on action fidelity, neglecting to consider how other factors such as affect and cognitions constrain perception and action behavior in sports practice (Pinder et al., 2015).

Researchers in psychology have demonstrated how task and environmental constraints shape the emergence of affective and cognitive responses (Pijpers et al., 2006; Oudejans and Nieuwenhuys, 2009). For example, Nieuwenhuys and Oudejans (2010) compared the behavior of police officers between two different practice tasks: a non-representative task where officers were required to shoot a ‘dummy’ target that could not move or shoot back versus a more representative task where the target could ‘shoot back.’ Practicing in the more representative task resulted in higher levels of anxiety and mental effort which were accompanied by poorer performance, quicker movement responses, increased blinking, and changes in postural orientation. Perception and action behaviors declined in the high anxiety task, raising questions about how to best train for tasks and environments that induce high amounts of affect.

An expanding body of work has demonstrated that enhancing the representativeness of practice tasks through the consideration of affective and cognitive demands will improve skill transfer to demanding environments (Oudejans and Pijpers, 2009, 2010; Nieuwenhuys and Oudejans, 2011; Alder et al., 2016). For instance, expert dart players who practiced under anxiety and high amounts of mental effort were able to maintain performance outcomes despite still experiencing high anxiety, arousal, and mental demands in a high anxiety transfer test (Oudejans and Pijpers, 2009). These findings suggest that training in conditions that simulate the affective and cognitive demands of performance environments may provide performers with opportunities to adapt to these performance constraints (Fajen, 2005; Oudejans and Nieuwenhuys, 2009; Rietveld and Kiverstein, 2014).

The importance of ensuring training environments simulate the affective-cognitive demands of performance environments has been captured in recent theoretical work. Affective learning design (ALD) builds on representative learning designs’ framework to consider affective and cognitive constraints in conjunction with environmental information (Headrick et al., 2015). Headrick et al. (2015, p. 85) advocate for practice tasks that afford “emotion-laden learning experiences that effectively simulate the constraints and demands of performance environments in sport.” The practical application of ALD promotes the design of practice tasks that afford rich competition-like experiences so that athletes are cognitively and affectively engaged so that they *think* and *feel* like they would in competition (Headrick et al., 2015; Pinder et al., 2015). Whilst work has examined affect and cognition in competition settings no studies have looked at whether typical sport training tasks simulate the affective and cognitive demands of competition and what the implications for skill transfer may be (Sève and Poizat, 2006; Hauw and Durand, 2007; Ria et al., 2011; Bridge et al., 2013).

At the elite level, fighting fellow squad members is a key training activity to prepare for combat competitions (Hodges and Starkes, 1996). Using the principle of fidelity, assessment of Taekwondo performance provides an opportunity to gain insight as to whether changes in affective and cognitive demands impact on performance behaviors in competition and training. One candidate performance variable that might be impacted is the interpersonal distance (IPD) of fighters (Dietrich et al., 2010). IPD is a global variable representative of the fighter–fighter system (Okumura et al., 2017). The distance between fighters’ provides different affordances for action and different striking techniques emerge and decay depending on this IPD (Hristovski et al., 2006; Okumura et al., 2017). Practically, IPD constrains the respective *attackability* of each fighter (i.e., specific critical IPDs invite an attack or being attacked). Given the influence of cognitive-affective subsystems on perception and action, any changes in affect should manifest in measures of IPD.

The aims of this study were twofold. First, we aimed to assess whether Taekwondo fighting in training adequately simulated the affective and cognitive demands of competition. Second, we wished to use the concept of fidelity to assess whether changes in these demands impacted the representativeness of fighting actions compared to competition. For our first aim it was hypothesized that the training environment would not adequately simulate the affective and cognitive demands of competition due to factors such as familiar opponents and lack of consequences. This would be evident in a reduction in affect (arousal, anxiety, and frequency of reported emotions) and less demanding cognition (mental effort and reported thoughts). In line with ALD, it was reasoned that this would lead to athletes being less emotionally and cognitively engaged in the task, creating intra and interpersonal fighting actions of lower fidelity. These reductions in engagement would manifest through a greater amount of time spent at larger IPD, larger attack initiation IPD, more predictable movement behavior, and fewer attacks.

## Materials and Methods

The university Human Research Ethics Committee of the first author approved the protocol for this study. All participants provided written informed consent prior to the commencement of the study in accordance with the Declaration of Helsinki.

### Participants

Ten international level senior Taekwondo athletes (seven male, three female) participated in the study. The average age of participants was 23 years (*SD* = 5 years). Participants were members of a national team and their demographics can be found in **Table 1**.

**Table 1:** Highest level of competition and world ranking range for each participant.

Participant	Highest level of competition	World ranking at testing
1	Olympics^∗^	5–10
2	Olympics^∗^	5–10
3	Olympics	11–20
4	World Championships^∗^	5–10
5	World Championships^∗^	11–20
6	World Championships^∗^	20–50
7	World Championships	100–150
8	G4 International competition^∗^	20–50
9	G2 International competition^∗^	100–150
10	G2 International competition^∗^	50–100

*^∗^Denotes multiple times competing at the highest level of competition.*

### Experimental Task

Data were collected during a national training camp. Participants were filmed and participated in mixed methods data collection as they fought in two distinct conditions – a typical training fight and a simulated competition fight. Training condition data was collected first during one of the national teams’ training sessions. The training condition consisted of the typical training activity of sparring against a fellow national team member. From practice observations, this is one of the teams most common practice tasks and would generally be prescribed multiple times per week. As per usual training custom, the coach acted as the referee and allocated fighters into pairs of similar ability. The composition of these pairs was determined according to the judgment of the national coach, who based the match ups on skill level, sex and weight category. Much like the competition task, the coach would usually provide instructional feedback to the athletes during the fight; however, he did so at his own discretion.

Competition condition data was subsequently collected during a ‘friendly’ competition against a visiting international team. This condition included competition-specific task constraints of an international opponent, crowd, professional referees, professional judges, and competition for an individual prize for highest score, and a team prize for most collective wins. In order to control for athletes intentions, in both conditions they were given the aim of winning the fight. Players received feedback from their coach at the coaches discretion just as they usually would in competition.

### Quantitative Measures

#### Perceived Anxiety and Arousal

Perceptions of cognitive and somatic anxiety were assessed using the Competitive State Anxiety Inventory-2 (Martens et al., 1990). Autonomic arousal was assessed by collecting the pre-fight average heart rate of participants in the 1 min epoch before the fight started. This approach has been used successfully before in similar studies to infer anxiety and arousal (Nieuwenhuys et al., 2012).

#### Perceived Mental Effort

Participants perception of mental effort has proved an insightful measure of task demands (e.g., Oudejans and Pijpers, 2009). Consequently, perceived mental effort was determined using the Rating Scale of Mental Effort (Zijlstra, 1993). This scale consists of a vertical axis scale with a range of 0–150 and descriptive anchors from not effortful to awfully effortful and has shown to be reliable across a range of real life settings (Zijlstra, 1993).

#### Movement Trajectories

To understand the emergent time-distance coordination strategies of fighters the evolution of system behavior was plotted over time for the entire fight. The movement trajectories of the players were manually tracked at 25 frames per second using digitizing software (Kinovea, version 0.8.25). This processes provided *x* and *y* coordinates for each participant across the duration of their fight. The court was calibrated using the known distances provided by the 1.00 m × 1.00 m mats that made up the 8.00 m × 8.00 m octagon fighting space. Digitizing consisted of tracking the center of mass, the mid-point between fighters’ feet. This was chosen due to past research that had used a similar technique in tracking individual movement trajectories (Headrick et al., 2012). Measurement accuracy was assessed by digitizing eight known distances within the calibrated space. The error of the measurement was found to be 0.02 m. The reliability of the digitizing methods was determined by re-digitizing the first round (2 min, or 33%) of a fight. This provided 3000 *x* and *y* coordinates for reliability analysis. The reliability between the two sets of *x* and *y* coordinates was assessed using an absolute agreement 2-way mixed effects intra class correlation coefficient (ICC) (Headrick et al., 2012). An acceptable degree of reliability was found: the average ICC for *x* coordinates was 0.994, 95% CI [0.994,0.995], and the average ICC for *y* coordinates was 0.997, 95% CI [0.994,0.998].

#### Interpersonal Distance

Interpersonal distance was determined using Pythagorean Theorem and the *x* and *y* coordinates for two fighters with the following calculation:

$$\mathrm{IPD}=\sqrt{{\left(x2-x1\right)}^{2}+{\left(y2-y1\right)}^{2}}$$

#### Attack Initiation Interpersonal Distance

Attack initiation IPD was analyzed using both the video and IPD data following previously published methods (Okumura et al., 2017). Attack initiation was determined using the video and defined as either the first forward movement of an attack, or if the athlete did not move forward, the time at which the foot first left the ground. Attack initiation IPD was defined as the IPD at the onset of attack initiation.

#### Number of Kicks

The number of attacks was assessed from the video data by counting the number of times participants performed a kicking action.

### Qualitative Measures

#### Self-confrontational Interview

Verbalisation data was collected from individual self-confrontational interviews with each participant using a course-of-action methodology (Theureau, 2003). Self-confrontational interviews are a tool used to ‘confront’ actors about their context specific behavior soon after that behavior took place and capture their in-performance cognitions and feelings (von Cranach and Harre, 1982). While watching a video replay of the fight, participants were asked to relive their experience and comment and/or answer questions based on what they did, thought, and felt during the fight (Theureau, 2003). These techniques reconstruct meaning actors give to their *in situ* activity through the recall and explanation of experiences (Ria et al., 2011). A number of previous studies have demonstrated how this approach is useful in understanding task demands and complementing quantitative approaches to increase understanding (Sève et al., 2005; Hauw and Durand, 2007; Seifert et al., 2017).

The interviews averaged 46 min in length (*SD* = 9 min) and were completed by the lead author who was familiar to the participants. To ensure trustworthiness of the data, leading questions that might have influenced the responses were avoided (Patton, 2002). During the interview both viewers could stop and rewind the video at any point. Generally, the video was stopped by either player or interviewer after an interaction between the two fighters. At this point the player would make a comment or the interviewer would ask a prompting question.

#### In-fight Emotions

Previous work has used the course-of-action methodology to determine in-competition emotions experienced by participants (Ria et al., 2011). During the confrontation interview participants were asked how they felt throughout the fight (Ria et al., 2011). Previous studies have shown that athletes are able to reliably recall their emotions in retrospect within 7 days (Martinent et al., 2012). To facilitate accurate recall of emotions, participants were provided with a list of emotions based on those reported in the Sports Emotion Questionnaire (SEQ), a 22 item tool developed to measure Emotions in sport (Jones et al., 2005). The list of emotions in the SEQ was developed from two sources: a list of emotions gathered from the literature, and completion of an open-ended questionnaire to identify emotions experienced by athletes in sport. The 22 items of the SEQ collapse into five basic emotions: happiness, anger, dejection, excitement, and anxiety. For the purposes of this study, collected emotions were collapsed into one of those five basic emotions.

### Procedure

A repeated measures design was adopted and the procedure for both conditions was identical. **Table 2** details the measures and their timing of collection. Upon arrival participants were fitted with heart-rate monitors (Firstbeat Technologies, Finland). Participants were then instructed to go about their usual warm-up routine before presenting to marshaling 10 min before the fight. At this point participants completed the Competitive State Anxiety Inventory-2. Participants then sat for 1 min before entering the ring to begin their fight. During this period pre-fight heart rate was collected. Fights consisted of three 2-min rounds, separated by a 1 min break. Official World Taekwondo Federation rules were adhered to and scoring was undertaken via the standard electronic protector and scoring system (Daedo TK-Strike, South Korea). Video data was collected using a digital video camera (Sony HXR-NX30P) positioned approximately 4.00 m above ground level, orientated at approximately 45 degrees to the central point of the court (Bartlett, 2007). This data was to be used to digitize player movement trajectories and as a stimulus for the confrontational interview. Following the fight, participants returned to the marshaling area to fill out the Rating Scale of Mental Effort. Within 24 h of the fight finishing participants completed the confrontational interview. None of the participants participated in another fight between data collection and their confrontational interview and were asked to avoid analyzing their fight.

**Table 2:** Table of measures and their timing of collection.

Measure	Pre-fight	Fight	Post-fight	24 h post-fight
Competitive State Anxiety Inventory-2	X			
Heart rate	X			
Video		X		
Rating Scale of Mental Effort			X	
Interview and in-fight emotions				X

### Quantitative Analysis

The predictability of participants’ movement trajectories was assessed by running the *x* and *y* coordinates of each participant in each condition through a sample entropy equation. The analysis for sample entropy was carried out using the R package RACMA (Borchers, 2017; R Core Team, 2017).

Interpersonal distance frequency and attack initiation IPD were analyzed descriptively by calculating the relative percentage of total observations that occurred in each 0.20 m IPD region between 0.00 m and 4.00 m in each condition (Okumura et al., 2017). The first zone was 0.00–0.20 m, the next zone 0.21–0.40 m, and so forth. For both variables (IPD frequency and attack initiation IPD) the 0.20 m IPD regions with the largest relative percentage of observations were selected for statistical comparison between conditions. These were called peak IPD frequency and peak attack initiation IPD.

Differences between competition and training conditions in perceived cognitive and somatic anxiety, mental effort, pre-fight heart rate, peak IPD frequency, peak attack initiation IPD and the number of kicks were analyzed using paired *t*-tests and Cohen’s d effect size calculations (Cohen, 1988). These were analyzed using SPSS computer software (version 19.0).

Differences between conditions for the entropy scores, attack initiation IPD, and in-fight emotion frequency were analyzed using linear mixed models, also performed in SPSS. The entropy mixed model had two fixed factors and one random factor; fixed factors: condition (training or competition) and coordinates (*x* or *y*), random factor: participant. The attack initiation IPD model had one fixed factor and one random factor; fixed factor: condition (training or competition), random factor: participant. The in-fight emotion frequency mixed model had two fixed factors and one random factor; fixed factors: condition (training or competition) and emotion (anger, anxiety, dejection, excitement, or happiness); random factor: participant. Significant effects were further investigated with pairwise comparisons using Bonferroni corrected alphas. Assumption testing of the residual values was carried out for all models and no violations were observed.

### Qualitative Analysis

The verbal data were analyzed using a four step methodology (Theureau, 2003; Gernigon and Arripe-longueville, 2004): (1) Producing a summary table of time-matched actions and verbal data, (2) Establishing the elementary units of meaning (EUM) for an individual, (3) Reconstructing the course of action for each EUM and labeling the EUM with a name representative of its content, (4) Grouping EUMs into like categories exclusive to either training or competition conditions (d’Arripe-Longueville et al., 2001; Theureau, 2003).

For the first step, two types of data were collated and paired chronologically: the verbatim transcripts from the confrontational interviews and match logs of the participants’ observed behavior during their fights. The second step consisted of identifying the smallest courses of action that were meaningful for each individual. For Taekwondo fighters this was generally confined to an interaction (attack or defense) with their opponent. The third step required identifying the underlying components of each elementary unit of meaning: the object, representment and interpretant (Hauw and Durand, 2007). This was achieved by asking a set of specific questions about the data: what is the participants’ intention (object)? What part of the situation is the athlete perceiving or making judgment of (representment)? And what prior knowledge is the athlete using to interpret the situation (interpretant)? An object is linked to a representment through an interpretant. When these components are linked together, an EUM emerges. The third step also included naming the EUM with a label representative of the contents (d’Arripe-Longueville et al., 2001; Gernigon and Arripe-longueville, 2004). EUMs were grouped into categories corresponding to higher order themes, which were then grouped into broader categories termed dimensions (d’Arripe-Longueville et al., 2001; Gernigon and Arripe-longueville, 2004). Summary labels were used for each grouping variable (d’Arripe-Longueville et al., 2001; Gernigon and Arripe-longueville, 2004). Finally we characterized the experience of the participants in competition and in training, specifically we were interested in the dimensions that lead to divergent experiences related to the affective and cognitive demands of each environments (Kiouak et al., 2016).

## Results

A summary of quantitative results can be found in **Table 3**.

**Table 3:** Results summary of perceived anxiety, arousal and perceived mental effort.

Variable	Training average	Competition average	*t* statistic	*p*	Mean difference	*SE* difference	Cohen’s *d*	95% Confidence Interval	
								Lower	Upper
CSAI-2 cognitive anxiety	15.2 ± 3.74	17.3 ± 4.35	3.99	0.003^∗^	2.1	0.53	1.26	0.91	3.29
CSAI-2 somatic anxiety	15.0 ± 3.83	17.8 ± 4.85	3.38	0.008^∗^	2.8	0.83	1.07	0.93	4.67
CSAI-2 confidence	24.7 ± 4.67	21.6 ± 4.60	-2.99	0.015^∗^	-3.1	1.04	-0.95	-5.45	-0.75
Rating Scale of Mental Effort	77.5 ± 27.87	102.5 ± 26.79	2.43	0.038^∗^	25	10.27	0.77	1.77	48.23
Ave pre-fight heart rate (BPM)	116.1 ± 7.06	129.0 ± 8.93	3.44	0.007^∗^	12.98	3.77	1.09	4.45	21.51
Number of kicks	55.8 ± 12.14	67.4 ± 13.23	2.57	0.03^∗^	11.6	4.51	0.81	1.40	21.80
Peak IPD frequency (cm)	187.0 ± 11.6	177.0 ± 8.23	-2.54	0.032^∗^	-10	3.94	-0.80	-18.92	-1.08
Peak attack initiation IPD (cm)	206.0 ± 18.97	188.0 ± 13.98	-3.86	0.004^∗^	-18	4.67	-1.22	-28.56	-7.44

*^∗^Denotes statistical significance (p < 0.05).*

### Perceived Anxiety and Arousal

**FIGURE 1:**
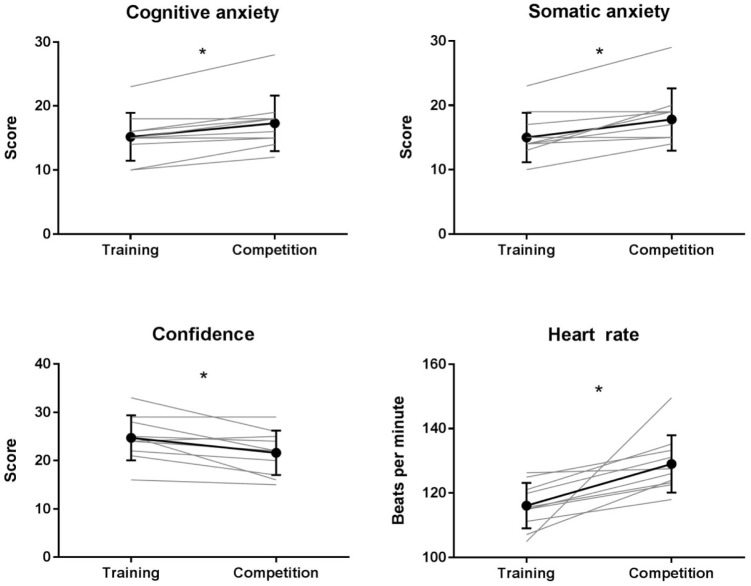
CSAI-2 factors: cognitive anxiety, somatic anxiety and confidence, and one minute pre-fight heart rate average for training and competition fights. Mean results and standard deviations are presented in bold, individual results are presented in light gray. ^∗^Indicates a significant difference between conditions (*p* < 0.05).

Perceived anxiety and arousal graphed results can be found in **Figure 1**. Greater levels of cognitive anxiety were reported in the competition condition (*M* = 17.3, *SD* = 4.35) compared to the training condition (*M* = 15.2, *SD* = 3.73); *t*(9) = 3.99, *p* < 0.05, *d* = 1.26.

Greater levels of somatic anxiety were reported in the competition condition (*M* = 17.8, *SD* = 4.85) than the training condition (*M* = 15.0, *SD* = 3.83); *t*(9) = 3.38, *p* < 0.05, *d* = 1.07.

Confidence levels were lower in competition (*M* = 21.6, *SD* = 4.60) compared to training (*M* = 24.70, *SD* = 4.67); *t*(9) = -2.99, *p* < 0.05, *d* = -0.95.

One minute pre-fight average heart rate was higher in competition (*M* = 129.0, *SD* = 8.93) compared to training (*M* = 116.1, *SD* = 7.10); *t*(9) = 3.44, *p* < 0.05, *d* = 1.09.

### Perceived Mental Effort

**FIGURE 2:**
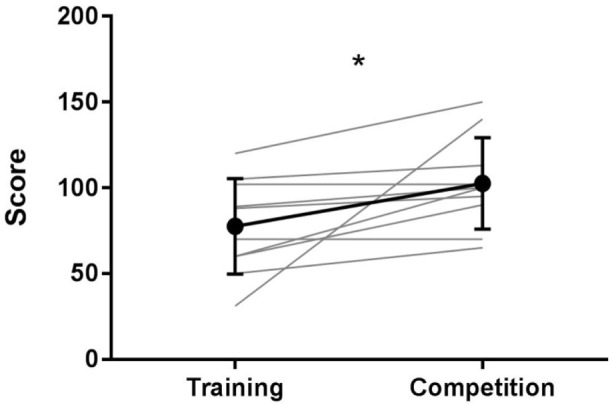
Rating scale of mental effort results for training and competition fights. Mean results and standard deviations are presented in bold, individual results are presented in light gray. ^∗^Indicates a significant difference between conditions (*p* < 0.05).

Fighters reported greater levels of mental effort (**Figure 2**) in the competition (*M* = 102.5, *SD* = 26.79) compared to the training condition (*M* = 77.5, *SD* = 27.87); *t*(9) = 2.43, *p* < 0.05, *d* = 0.77.

### Movement Trajectories

**FIGURE 3:**
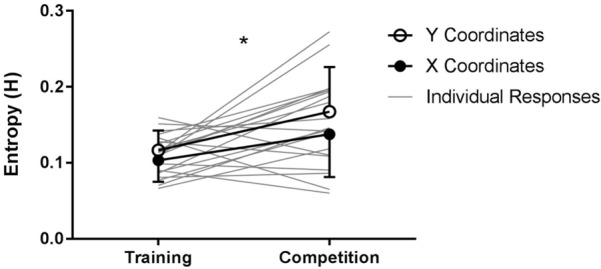
Predictability of movement trajectories assessed using sample entropy (H) for training and competition fights. Mean results and standard deviations are presented in bold for both x and y coordinates, individual results are presented in light gray. ^∗^Indicates a significant main effect for condition (*p* < 0.05).

The linear mixed model revealed a significant fixed effect for condition, *F*(1,28) = 12.408, *p* = 0.001 (**Figure 3**). *Post hoc* pairwise comparisons revealed that the movement trajectories of participants were more unpredictable in competition (*M* = 0.15, *SD* = 0.06) compared to training (*M* = 0.11, *SD* = 0.03), *p* = 0.001, 95% CI [0.01, 0.07], *d* = 0.84. There was no significant effect for coordinates *F*(1,28) = 3.18, *p* = 0.085.

### Interpersonal Distance Frequency

**FIGURE 4:**
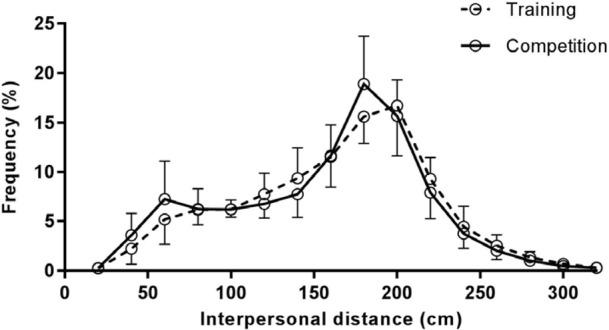
Percentage of time spent at interpersonal distances in training and competition fights. Interpersonal distances are presented in 0.20 m bins.

The percentage scores for time spent at each IPD (**Figure 4**) reveal that the peak region of IPD frequency was closer in competition (*M* = 177.0 cm, *SD* = 8.23) compared to training (*M* = 187.0 cm, *SD* = 11.6); *t*(9) = -2.45, *p* < 0.05, *d* = -0.80.

### Attack Initiation Interpersonal Distance

**FIGURE 5:**
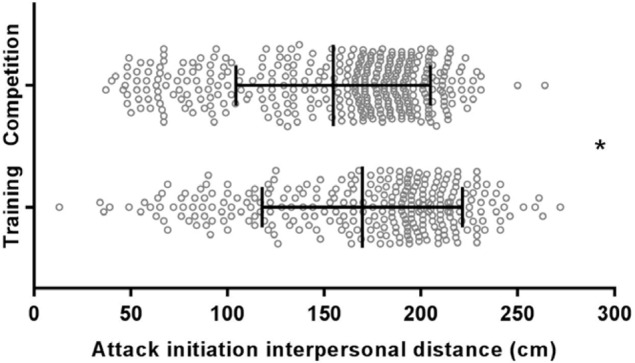
Interpersonal distance of all attacks initiated in training and competition fights. Mean results and standard deviations are presented in bold, individual attacks are presented in light gray circles. The mean and standard deviation is presented. ^∗^Indicates a significant fixed effect of condition (*p* < 0.05).

The linear mixed model revealed a significant fixed effect for condition *F*(1,981.77) = 10.631, *p* = 0.001. *Post hoc* pairwise comparisons revealed that attack initiation IPD was closer in competition (*M* = 156.87, *SD* = 47.25) compared to training (*M* = 166.62, *SD* = 48.70), *p* = 0.001, 95% CI [-16.088, -3.999], *d* = -0.203. These results can be found graphed in **Figure 5**.

**FIGURE 6:**
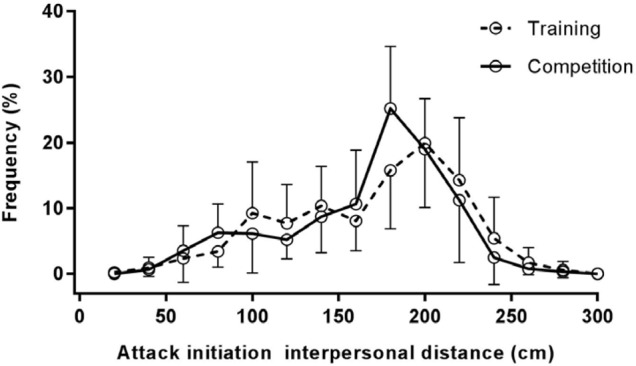
Percentage of attacks initiated from interpersonal distances in training and competition fights. Interpersonal distances are presented in 0.20 m bins.

Analysis of the peak IPD zone of attack (**Figure 6**) was closer in competition (*M* = 188.0 cm, *SD* = 13.98) compared to training (*M* = 206.0 cm, *SD* = 18.97); *t*(9) = -3.86, *p* < 0.05, *d* = -1.22.

### Number of Kicks

The number of kicks was greater in competition (*M* = 67.4, *SD* = 13.23) compared to training (*M* = 55.8, *SD* = 12.14); *t*(9) = 2.57, *p* < 0.05, *d* = 0.81.

### Self-Confrontational Interview

Self-confrontational interview data can be seen summarized in **Tables 4**, **5**.

**Table 4:** Synthesized interview data from the competition condition relating to affective-cognitive differences between environments.

	Competition	
Dimensions	Themes	EUM examples
Arousal (20 EUMs)	High individual arousal	Feel ‘switched on’ and ready to fight
		Feeling fast
	High fight intensity	Defend high intensity attack from opponent
		Lift fight intensity to match opponent
		
Mental challenge (38 EUMs)	Problem solving	Thinking about tactics/techniques that might be useful
		Hypothesis test possible tactical/technical solution
	Opponent unfamiliarity	Surprised by opponents actions
		Unsure what tactics/techniques will be successful
	Difficulty executing own techniques/tactics	Difficulty executing technique or tactic
		Opponent able to absorb attack
		
Pressure (38 EUMs)	Task pressure	Under pressure due to position on the court
		Under pressure due to the score
	Opponent pressure	Feel uncomfortable due to the aggressive nature of opponent
		Concerned about head kick from opponent

**Table 5:** Synthesized interview data from the training condition relating to affective-cognitive differences between environments.

	Training	
Dimensions	Themes	EUM examples
Low arousal (27 EUMs)	Low individual arousal	Unsuccessfully attempt to enhance arousal level
		Feeling sluggish
	Low fight intensity	Low intensity attack from opponent
		Avoiding engagement
		
Low mental challenge (33 EUMs)	Use established knowledge of opponent	Select tactic/technique based on prior knowledge of opponent
		Anticipate opponents behavior based on prior knowledge
	Not challenged by opponent	Able to absorb opponents attack
		Have established attack/defense solution ready

### In-fight Emotions

**FIGURE 7:**
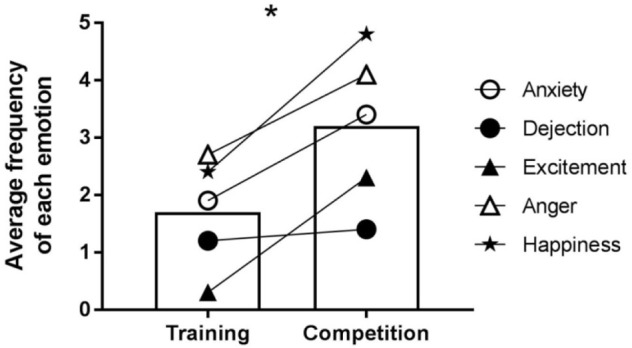
Average frequency of each recalled in-fight emotions for training and competition fights. ^∗^Indicates a significant fixed effect for condition.

**FIGURE 8:**
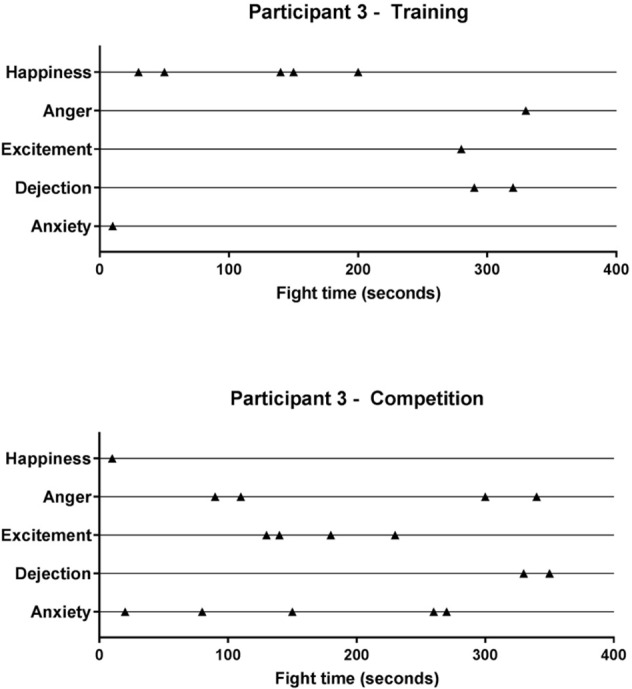
Exemplar data for the temporal arrangement of recalled in-fight emotions for training and competition fights.

In-fight emotion frequency results are summarized in **Figure 7**, while exemplar data is provided in **Figure 8**. Results of the linear mixed model revealed no significant interaction between emotion and condition. There was, however, a significant fixed effect of emotion, *F*(4,81) = 7.141, *p* = 0.000, and a significant fixed effect for condition, *F*(1,81) = 16.363, *p* = 0.000. *Post hoc* pairwise comparisons revealed that the mean frequency of each emotion was greater in competition (*M* = 3.20, *SD* = 2.39) compared to training (*M* = 1.70, *SD* = 1.71), *p* = 0.000, 95% CI [0.76, 2.24], *d* = 0.63.

## Discussion

Research has focused on the role of physical information when designing representative learning environments, yet has neglected the role of affect and cognition and how they might influence the representativeness of behavior (Pinder et al., 2011a; Headrick et al., 2015). The aims of the study were to assess whether Taekwondo fighting in training adequately simulates the affective and cognitive demands of competition and secondly whether the affective-cognitive demands observed in training impact on the representativeness of individual and interpersonal behavior relative to competition.

When fighting in training, participants reported lower levels of anxiety, arousal. and mental effort and reported different goals, suggesting that fighting in training does not recreate the cognitive and affective demands of competition. These decreased demands were associated with individual and interpersonal behavior of lower fidelity. In training, individual fighters performed fewer kicks and attacked from further away, whilst the fighter–fighter system generated larger IPDs. The data show reductions in cognitive and affective demands are associated with different individual and interpersonal fighting behavior in training. The discussion will first cover each factor individually (affect, cognition, and behavior) before discussing possible interactions between the three and the implications for the design of representative learning environments and skill transfer.

### The Affective Demands of Training

The first aim of this study involved comparing the affective demands of fighting in training relative to competition. Results from the perceptual scales, interviews and pre-fight maximum heart rate were all congruent: fighting in training has reduced affective demands relative to competition. The triangulation of these results suggests that fighting in training alone does not afford similar levels of arousal and anxiety as fighting in competition. Exemplar interview data reveals the extent of this issue with one fighter: “This is a common problem for me. I’m not very stimulated and I’m in a bad mood. Whenever I’m fighting<players of own nationality> I struggle to get stimulated – I am not challenged.” And: “I’m not in the zone, this isn’t how I would want to feel in competition.” This finding is in line with previous work that has demonstrated differences in arousal and anxiety between training and performance environments (Haneishi et al., 2007; Bridge et al., 2013; Fernandez-fernandez et al., 2015).

During the training fights participants reported a reduced frequency of emotions. These results support the dynamic nature of emotions in sport which suggests emotions emerge and decay based on performance situations (Cerin et al., 2000; Hanin, 2003; Ria et al., 2011; Martinent et al., 2012). One of the key practical applications of ALD is the need to design training tasks that emotionally engage athletes regardless of valence. The reduced number of emotions experienced by athletes in training suggests that fighting in training may not be as engaging compared to competition, perhaps due to absence of stimulating competition-factors like prizes, judges and a crowd. Overall, these results may have implications for the transfer of skills between performance settings. Learning to cope with emotions created by performance environments such as competition can be as important as a learning technique (Pinder et al., 2015). Research assessing the affective demands of learning environments shows that superior transfer of performance is observed when the practice environment closely simulates these demands (Nieuwenhuys et al., 2009; Nieuwenhuys and Oudejans, 2011).

### The Cognitive Demands of Training

The current results suggest that training fights were less cognitively demanding compared to competition. This was evident in participants’ perceptions of mental effort, which was significantly lower in the training fight. Further, a dimension related to mental challenge emerged from the interview data in both training and competition. In training participants reported a low mental challenge as they used prior knowledge of their opponent to aid their own action selection and to predict what their opponent would do. For instance, one participant mentioned “If I push him on the back foot he will do something stupid. He doesn’t have a good left leg under so I know I can attack. I know his game and what he’s trying to do.” Contrastingly, in competition, participants were less familiar with their opponents so spent time determining what their opponent was trying to do. “I’m trying to get him to move backward. I’m cutting^2^ and he’s not moving. I’m thinking what’s going on? Normally if I cut, he should move back, but he’s not. So I’m trying to process the whole thing and I’m thinking I need to change my tactics.” These results confirm previous work on in-competition courses of action which showed table tennis players spent time constructing and validating knowledge of their opponent and strive to build a model of their opponents weaknesses and intentions (Sève and Poizat, 2006). Our findings extend this literature by showing that in training against familiar opponents, players are less likely to cognitively problem solve compared to when they are fighting unfamiliar opponents in competition. In the future it would be interesting to examine whether these changes in cognitive demands would still be observed when players are fighting a familiar opponent in competition. Overall, the triangulation of these results suggests that simply fighting in training is not as cognitively demanding as fighting in competition. This has potential implications for training design, where tasks should be appropriately challenging to the individual to facilitate skill learning (Guadagnoli and Lee, 2004).

### Individual and Interpersonal Behavior

The second aim of this paper was to assess whether the affective and cognitive demands of the training environment were associated with changes in the fidelity of individual and interpersonal fighting behavior. Behaviors are of low fidelity when they are not representative of those observed in a reference environment (Stoffregen, 2007). When behavior in training tasks is of low fidelity compared to competition, it is likely to compromise the transfer potential of sporting skills (Pinder et al., 2009; Barris et al., 2013). The results of this study reveal that the individual and interpersonal actions of the fighters were different in training. In training, participants kicked less, initiated their attacks from further away and displayed more predictable movement displacement. The interpersonal coordination of fighters was also different as the fighter–fighter system generated larger IPDs.

The larger IPDs generated by the fighter–fighter system in this study would suggest that different actions are afforded and supported in training. In the combat sports, action selection is based on the scaled distance between a striker and their target (Hristovski et al., 2006). Certain distances afford and support specific striking actions. For instance, intermediate IPDs encourage flexible behaviors by affording a greater variety of striking actions (Hristovski et al., 2006). However, at larger IPDs (those approaching and exceeding an individual’s maximum reach) fewer actions are afforded, and at a critical distance, no striking actions are supported (Hristovski et al., 2006). At these larger IPDs, athletes exhibit less flexible action solutions, perhaps explaining why fewer kicks were recorded in the training environment. Simply put, the distances that fighters spent their time at in training does not afford the same number of actions as the closer distances in competition did, nor does it afford players as many opportunities to develop the flexible action solutions required at smaller IPDs.

These differences may also have implications for perceptual attunement and the way learners educate their attention (Michaels and Jacobs, 2007). A key aspect of learning is attuning to the most useful sources of information to support the selection and control of action (Fajen et al., 2009). As learners progress, the information they use evolves in a Darwinian sense as more useful sources of information are identified (Michaels and Jacobs, 2007). Therefore, if participants spend their time at larger IPDs, they may not be afforded opportunities to attune to the most useful sources of information. The results of this study suggest that when fighting in training, Taekwondo athletes are not placed under the same levels of perceptual stress as in competition, where they are forced to co-adapt to opponents movements which are more unpredictable and occur at closer distances. This has possible negative implications for transfer given that players are not practicing adapting to opponents at IPD representative of competition.

These results highlight how emergent behavior may be shaped by a complex interaction between affect, cognition, and action (Headrick et al., 2015). For instance, behavior in the training environment is associated with lower levels of arousal and anxiety interacting with reduced cognitive demands to constrain the type of fighting behavior that was observed: fewer attacks and more time spent at IPDs further away from their opponent. These results align with earlier work in sport, which highlights how changes in affect constraints the way people perceive and act within the world (Pijpers et al., 2006). An ecological dynamics approach would suggest that learning is the product of continued agent–environment interactions that lead to the emergence of functional patterns of behavior (Fajen and Warren, 2003). This means that sportspeople adapt to the environment and social situations they find themselves participating in (Oudejans and Pijpers, 2009; Rietveld and Kiverstein, 2014). This highlights the importance of designing practice simulations that adequately represent the affective and cognitive constraints and demands of the competition environment.

### Implications for Training Design and Transfer

These findings highlight a limitation of the focus on preserving or simulating perceptual information from competition to enhance skill learning and transfer (Pinder et al., 2011b). Previous work has focused largely on the information stimulus and action responses of learners; however, these results suggest that practice design is a complex issue and requires consideration of other factors such as affect and cognition (Pinder et al., 2015). For instance, fighting in training satisfies principles of representative design as it is predicated upon the same ‘information’ (i.e., another opponent) as the competition environment. However, when fighting in training, Taekwondo athletes are clearly solicited by a different field of affordances, which is evident in the low fidelity action responses. To ensure transfer it has been suggested that training tasks should be assessed not by the representativeness of information, but instead by the affordances on offer and the performances they support (Araujo and Davids, 2015). Araujo and Davids (2015) argued that behavior of lower fidelity is acceptable if it ‘emerges under the constraints of the competitive performance environment. However, for this to be true, our data suggests we may need to also consider not just the informational properties, but the affective and cognitive constraints and demands (Headrick et al., 2015).

One way to sample affordances that solicit representative action, cognitive and affective responses is through following a principled approach such as ALD (Headrick et al., 2015). One of the claims of ALD is that practitioners need to sample, predict and plan for the potential affective and cognitive circumstances in competition. Practically, ALD suggests creating scenarios and vignettes sampled from the competitive environment so that athletes think, feel, and act like they would in competition (Headrick et al., 2015). Therefore, sampling the affordances that consider affective and cognitive demands from the performance environment is an important principle that should be satisfied for the transfer of behavior between settings (Araujo and Davids, 2015).

## Conclusion

This study showed that fighting in training does not adequately simulate the affective and cognitive demands of fighting in competition. These reduced demands are associated with individual and interpersonal behavior of low fidelity relative to competition. Therefore, we highlight the importance of considering the often overlooked aspects of affect and cognition when designing representative practice environments. Simply fighting in training does not simulate the constraints and demands of fighting in competition due to lower levels of anxiety and arousal, decreased mental challenge, and different movement behavior. Consequently, this is likely to negatively impact on skill transfer from training to competition.

## Author Contributions

MM, DF, IR, JH, and DM contributed to the design of the work. MM and DM acquired the data. MM, DF, IR, and JH contributed to the interpretation of the data. MM drafted the work. MM, DF, IR, JH, and DM revised the work critically. MM, DF, IR, JH, and DM approved the final version to be published. MM, DF, IR, JH, and DM agreed to be accountable for all aspects of the work.

## Conflict of Interest Statement

The authors declare that the research was conducted in the absence of any commercial or financial relationships that could be construed as a potential conflict of interest.

## Footnotes

1 The term ‘affect’ will be used to refer to a range of phenomena such as feelings, emotions and mood. The terms affect and emotion will be used interchangeably to follow previous work in the area (Lewis and Granic, 2000; Headrick et al., 2015).

2 The cut kick is a Taekwondo kicking technique.

## References

[B1] Alder D. Ford P. R. Causer J. Williams A. M. (2016). The effects of high- and low-anxiety training on the anticipation judgments of elite performers. *J. Sport Exerc. Psychol.* 38 93–104. 10.1123/jsep.2015-0145 27018561

[B2] Araujo D. Davids K. (2015). Towards a theoretically-driven model of correspondence between behaviours in one context to another: implications for studying sport performance. *Int. J. Sport Psychol.* 46 745–757. 10.7352/IJSP

[B3] Araujo D. Davids K. W. Passos P. (2007). Ecological validity, representative design, and correspondence between experimental task constraints and behavioral setting: comment on Rogers, Kadar, and Costall (2005). *Ecol. Psychol.* 19 69–78. 10.1080/10407410709336951

[B4] Barris S. Davids K. Farrow D. (2013). Representative learning design in springboard diving: is dry-land training representative of a pool dive? *Eur. J. Sport Sci.* 13 638–645. 10.1080/17461391.2013.770923 24251741

[B5] Barris S. Farrow D. Davids K. (2014). Increasing functional variability in the preparatory phase of the takeoff improves elite springboard diving performance. *Res. Q. Exerc. Sport* 85 97–106. 10.1080/02701367.2013.872220 24749241

[B6] Bartlett R. (2007). *Introduction to Sports Biomechanics: Analysing Human Movement Patterns*. London: Routledge.

[B7] Borchers H. W. (2017). *pracma: Practical Numerical Math Functions*. Available at: https://CRAN.R-project.org/package=pracma

[B8] Bridge C. A. Mcnaughton L. R. Close G. L. Drust B. Kingdom U. Sciences E. (2013). Taekwondo exercise protocols do not recreate the physiological responses of championship combat. *Int. J. Sports Med.* 34 573–581. 10.1055/s-0032-1327578 23296399

[B9] Bruineberg J. Rietveld E. (2014). Self-organization, free energy minimization, and optimal grip on a field of affordances. *Front. Hum. Neurosci.* 8:599. 10.3389/fnhum.2014.00599 25161615 PMC4130179

[B10] Brunswik E. (1956). *Perception and the Representative Design of Psychological Experiments*, 2nd Edn. Berkeley, CA: University of California Press.

[B11] Cerin E. Szabo A. Hunt N. Williams C. (2000). Temporal patterning of competitive emotions: a critical review. *J. Sports Sci.* 18 605–626. 10.1080/02640410050082314 10972411

[B12] Cohen D. (1988). *Statistical Power Analysis for the Behaviour Science*, 2nd Edn. Hillsdale, MI: Erlbaum.

[B13] d’Arripe-Longueville F. Saury J. Fournier J. Durand M. (2001). Coach-athlete interaction during elite archery competitions: an application of methodological frameworks used in ergonomics research to sport psychology. *J. Appl. Sport Psychol.* 13 275–299. 10.1080/104132001753144419

[B14] Davids K. Araújo D. (2010). The concept of ‘Organismic Asymmetry’ in sport science. *J. Sci. Med. Sport* 13 633–640. 10.1016/j.jsams.2010.05.002 20580313

[B15] Davids K. Araújo D. Hristovski R. (2012). “Ecological dynamics and motor learning design in sport,” in *Skill Acquisition in Sport: Research, Theory and Practice* eds Williams A. M. Hodges N. J. (Abingdon: Routledge) 112–129.

[B16] Dicks M. Button C. Davids K. (2010). Examination of gaze behaviors under in situ and video simulation task constraints reveals differences in information pickup for perception and action. *Atten. Percept. Psychophys.* 72 706–720. 10.3758/APP 20348577

[B17] Dietrich G. Bredin J. Kerlirzin Y. (2010). Interpersonal distance modeling during fighting activities. *Motor Control* 14 509–527. 21051791 10.1123/mcj.14.4.509

[B18] Fajen B. R. (2005). Perceiving possibilities for action: on the necessity of calibration and perceptual learning for the visual guidance of action. *Perception* 34 717–740. 10.1068/p5405 16042193

[B19] Fajen B. R. Riley M. Turvey M. (2009). Information, affordances, and the control of action in sport. *Int. J. Sport Psychol.* 40 79–107.

[B20] Fajen B. R. Warren W. H. (2003). Behavioral dynamics of steering, obstacle avoidance, and route selection. *J. Exp. Psychol. Hum. Percept. Perform.* 29 343–362. 10.1167/1.3.184 12760620

[B21] Fernandez-fernandez J. Boullosa D. A. Sanz-Rivas D. Abreu L. Filaire E. Mendez-Villanueva A. (2015). Psychophysiological stress responses during training and competition in young female competitive tennis players. *Int. J. Sports Med.* 36 22–28. 10.1055/s-0034-1384544 25251448

[B22] Gernigon C. Arripe-longueville F. (2004). A dynamical systems perspective on goal involvement states in sport. *J. Sport Exerc. Psychol.* 26 572–596. 20587824

[B23] Gibson J. J. (1979). *Ecological Approach to Visual Perception*, 6th Edn. Boston, MA: Houghton Mifflin.

[B24] Greenwood D. Davids K. Renshaw I. (2016). The role of a vertical reference point in changing gait regulation in cricket run-ups. *Eur. J. Sport Sci.* 16 794–800. 10.1080/17461391.2016.1151943 26902778

[B25] Guadagnoli M. A. Lee T. D. (2004). Challenge point: a framework for conceptualizing the effects of various practice conditions in motor learning. *J. Mot. Behav.* 36 212–224. 10.3200/JMBR.36.2.212-224 15130871

[B26] Haneishi K. Fry A. C. Moore C. A. Schilling B. K. Li Y. Fry M. D. (2007). Cortisol and stress responses during a game and practice in female collegiate soccer players. *J. Strength Cond. Res.* 21 583–588. 10.1519/R-b20496.1 17530979

[B27] Hanin Y. L. (2003). Performance related emotional states in sport: a qualitative analysis. *Forum Qual. Soc. Res.* 4:5.

[B28] Hauw D. Durand M. (2007). Situated analysis of elite trampolinists’ problems in competition using retrospective interviews. *J. Sports Sci.* 25 173–183. 10.1080/02640410600624269 17127592

[B29] Headrick J. Davids K. Renshaw I. Araújo D. Passos P. Fernandes O. (2012). Proximity-to-goal as a constraint on patterns of behaviour in attacker–defender dyads in team games. *J. Sports Sci.* 30 247–253. 10.1080/02640414.2011.640706 22176036

[B30] Headrick J. Renshaw I. Davids K. Pinder R. Araujo D. (2015). The dynamics of expertise acquisition in sport: the role of affective learning design. *Psychol. Sport Exerc.* 16 83–90.

[B31] Hodges N. J. Starkes J. L. (1996). Wrestling with the nature expertise: a sport specific test of Ericsson, Krampe and Tesch-Romer’s (1993) theory of “deliberate practice. *Int. J. Sport Psychol.* 27 400–424.

[B32] Hristovski R. Davids K. Button C. (2006). How boxers decide to punch a target: emergent behaviour in nonlinear dynamical movement systems. *J. Sport Sci. Med.* 5 60–73. 24357978 PMC3863932

[B33] Jones M. Lane A. M. Bray S. R. Uphill M. Catlin J. (2005). Development and validation of the sport emotion questionnaire. *J. Sport Exerc. Psychol.* 27 407–431.

[B34] Kelso J. A. S. (1995). *Dynamic Patterns: The Self-Organisation of Brain and Behaviour*. Cambridge: MIT Press.

[B35] Kiouak M. R. Saury J. Durand M. Bourbousson J. (2016). Joint action of a pair of rowers in a race: shared experiences of effectiveness are shaped by interpersonal mechanical states. *Front. Psychol.* 7:720. 10.3389/fpsyg.2016.00720 27242628 PMC4870391

[B36] Lewis M. D. Granic I. (2000). *Emotion, Development, and Self-Organization: Dynamic Systems Approaches to Emotional Development*. Cambridge: Cambridge University Press.

[B37] Martens R. Vealy R. Burton D. Bump L. Smith D. (1990). “Development and validation of the competitive sports anxiety inventory 2” in *Competitive Anxiety in Sport* eds Martens R. Vealey R. Burton D. (Champaign, IL: Human Kinetics) 117–178.

[B38] Martinent G. Campo M. Ferrand C. (2012). A descriptive study of emotional process during competition: nature, frequency, direction, duration and co-occurrence of discrete emotions. *Psychol. Sport Exerc.* 13 142–151. 10.1016/j.psychsport.2011.10.006

[B39] Michaels C. F. Jacobs D. M. (2007). Direct learning. *Ecol. Psychol.* 19 321–349.

[B40] Nieuwenhuys A. Caljouw S. R. Leijsen M. R. Schmeits B. A. J. Oudejans R. R. D. (2009). Quantifying police officers’ arrest and self-defence skills: does performance decrease under pressure? *Ergonomics* 52 1460–1468. 10.1080/00140130903287981 19941180

[B41] Nieuwenhuys A. Canal-Bruland R. Oudejans R. R. D. (2012). Effects of threat on police officers’ shooting behavior: anxiety, action specificity, and affective influences on perception. *Appl. Cogn. Psychol.* 26 608–615. 10.1002/acp.2838

[B42] Nieuwenhuys A. Oudejans R. R. D. (2010). Effects of anxiety on handgun shooting behavior of police officers: a pilot study. *Anxiety Stress Coping* 23 225–233. 10.1080/10615800902977494 19462309

[B43] Nieuwenhuys A. Oudejans R. R. D. (2011). Training with anxiety: short- and long-term effects on police officers’ shooting behavior under pressure. *Cogn. Process* 12 277–288. 10.1007/s10339-011-0396-x 21431863 PMC3142543

[B44] Okumura M. Kijima A. Yamamoto Y. (2017). Perception of affordances for striking regulates interpersonal distance maneuvers of intermediate and expert players in kendo matches. *Ecol. Psychol.* 29 1–22. 10.1080/10407413.2017.1270147

[B45] Oudejans R. R. D. Nieuwenhuys A. (2009). Perceiving and moving in sports and other high-pressure contexts. *Prog. Brain Res.* 174 35–48. 10.1016/S0079-6123(09)01304-1 19477328

[B46] Oudejans R. R. D. Pijpers J. R. (2009). Training with anxiety has a positive effect on expert perceptual-motor performance under pressure. *Q. J. Exp. Psychol.* 62 1631–1647. 10.1080/17470210802557702 19123115

[B47] Oudejans R. R. D. Pijpers J. R. (2010). Training with mild anxiety may prevent choking under higher levels of anxiety. *Psychol. Sport Exerc.* 11 44–50. 10.1016/j.psychsport.2009.05.002

[B48] Patton M. (2002). *Qualitative Research and Evaluation Methods*, 3rd Edn. London: Sae Publications.

[B49] Pijpers J. R. Oudejans R. R. D. Bakker F. C. Beek P. J. (2006). The role of anxiety in perceiving and realizing affordances. *Ecol. Psychol.* 18 37–41. 10.1207/s15326969eco1803

[B50] Pinder R. Davids K. Renshaw I. Araújo D. (2011a). Manipulating informational constraints shapes movement reorganization in interceptive actions. *Atten. Percept. Psychophys.* 73 1242–1254. 10.3758/s13414-011-0102-1 21327746

[B51] Pinder R. Davids K. Renshaw I. Araújo D. (2011b). Representative learning design and functionality of research and practice in sport. *J. Sport Exerc. Psychol.* 33 146–155. 21451175 10.1123/jsep.33.1.146

[B52] Pinder R. Headrick J. Oudejans R. (2015). “Issues and challenges in developing representative tasks in sport,” in *Routledge Handbook of Sport Expertise,” in the Routledge Handbook of Sports Expertise* eds Baker J. Farrow D. (London: Routledge) 269–281.

[B53] Pinder R. Renshaw I. Davids K. (2009). Information-movement coupling in developing cricketers under changing ecological practice constraints. *Hum. Mov. Sci.* 28 468–479. 10.1016/j.humov.2009.02.003 19339072

[B54] R Core Team R. (2017). *R: A Language and Environment for Statistical Computing*. Vienna: R Core Team.

[B55] Ria L. Poizat G. Saury J. Durand M. (2011). Performance-induced emotions experienced during high-stakes table tennis matches. *Psychol. Sport Exerc.* 8 25–46.

[B56] Rietveld E. Kiverstein J. (2014). A rich landscape of affordances. *Ecol. Psychol.* 26 325–352. 10.1080/10407413.2014.958035

[B57] Seifert L. Lardy J. Bourbousson J. Adé D. Nordez A. Thouvarecq R. (2017). Interpersonal coordination and individual organization combined with shared phenomenological experience in rowing performance: two case studies. *Front. Psychol.* 8:75. 10.3389/fpsyg.2017.00075 28194127 PMC5278567

[B58] Sève C. Poizat G. (2006). A grounded theory of elite male table tennis players’ activity during matches. *Sport Psychol.* 20 58–73.

[B59] Sève C. Saury J. Leblanc S. Durand M. (2005). Course-of-action theory in table tennis: a qualitative analysis of the knowledge used by three elite players during matches. *Rev. Eur. Psychol. Appl.* 55 145–155. 10.1016/j.erap.2005.04.001

[B60] Shim J. Carlton L. G. Chow J. W. Chae W.-S. (2005). The use of anticipatory visual cues by highly skilled tennis players. *J. Mot. Behav.* 37 164–175. 10.3200/JMBR.37.2.164-175 15730949

[B61] Stoffregen T. A. (2007). “Action fidelity,” in *Enaction and Enactive Interfaces: A Handbook of Terms* eds Luciani A. Cadoz C. (Grenoble: Enactive Systems Books) 328.

[B62] Stoffregen T. A. Bardy G. B. Smart L. J. Pagulayan R. J. (2003). “On the nature and evaluation of fidelity in virtual environments,” in *Virtual and Adaptive Enviroments: Applications, Implications and Human Performance Issues* eds Hettinger L. J. Haas M. W. (Mahwah, NJ: Lawrence Erlbaum Associates) 111–128.

[B63] Theureau J. (2003). “Course-of-action analysis and course-of-action centred design,” in *Handbook of Cognitive Task Design* ed. Hollnagel E. (Mahwah, NJ: Lawrence Erlbaum Associates).

[B64] von Cranach M. Harre R. (eds) (1982). *The Analysis of Action: Recent Theoretical and Empirical Advances*. Cambridge: Cambridge University Press.

[B65] Zijlstra F. (1993). *Efficiency in work Behaviour: A Design Approach for Modern Tools*. Amsterdam: Delft University Press.

